# Adverse prognosis gene expression patterns in metastatic castration‐resistant prostate cancer

**DOI:** 10.1002/1878-0261.70001

**Published:** 2025-02-22

**Authors:** Marina N. Sharifi, Eric Feng, Nicholas R. Rydzewski, Amy K. Taylor, Jamie M. Sperger, Yue Shi, Kyle T. Helzer, Matthew L. Bootsma, Viridiana Carreno, Alex H. Chang, Luke A. Nunamaker, Grace C. Blitzer, Tianfu Andy Shang, Aishwarya Subramanian, Anders Bjartell, Andreas Josefsson, Pernilla Wikström, Emily Feng, Manish Kohli, Rendong Yang, Scott M. Dehm, Eric J. Small, Rahul Aggarwal, David A. Quigley, Joshua M. Lang, Shuang G. Zhao, Martin Sjöström

**Affiliations:** ^1^ Carbone Cancer Center University of Wisconsin‐Madison WI USA; ^2^ Department of Medicine University of Wisconsin‐Madison WI USA; ^3^ Helen Diller Family Comprehensive Cancer Center University of California, San Francisco CA USA; ^4^ Department of Radiation Oncology University of California, San Francisco CA USA; ^5^ Department of Human Oncology University of Wisconsin‐Madison WI USA; ^6^ Department of Translational Medicine Lund University Malmö Sweden; ^7^ Department of Urology Skåne University Hospital Malmö Sweden; ^8^ Department of Diagnostics and Interventions, Urology Umeå University Sweden; ^9^ Wallenberg Center for Molecular Medicine Umeå University Sweden; ^10^ Department of Medical Biosciences, Pathology Umeå University Sweden; ^11^ Huntsman Cancer Institute University of Utah Salt Lake City UT USA; ^12^ Department of Urology Northwestern University Feinberg School of Medicine Chicago IL USA; ^13^ Masonic Cancer Center University of Minnesota Minneapolis MN USA; ^14^ Department of Laboratory Medicine and Pathology University of Minnesota Minneapolis MN USA; ^15^ Department of Urology University of Minnesota Minneapolis MN USA; ^16^ Division of Hematology and Oncology, Department of Medicine University of California, San Francisco CA USA; ^17^ Department of Urology University of California, San Francisco CA USA; ^18^ Department of Epidemiology and Biostatistics University of California, San Francisco CA USA; ^19^ William S. Middleton Memorial Veterans' Hospital Madison WI USA; ^20^ Division of Oncology, Department of Clinical Sciences Lund Lund University Sweden; ^21^ Department of Hematology, Oncology, and Radiation Physics Skåne University Hospital Lund Sweden

**Keywords:** biomarker, gene expression, metastatic castration‐resistant prostate cancer, precision medicine, prognosis

## Abstract

Metastatic castration‐resistant prostate cancer (mCRPC) is a heterogeneous disease. Several studies have identified transcriptional subtypes of mCRPC, but comprehensive analysis of prognostic gene expression pathways has been limited. Therefore, we aggregated a cohort of 1012 mCRPC tissue samples from 769 patients and investigated the association of gene expression‐based pathways with clinical outcomes and intrapatient and intratumor heterogeneity. Survival data were obtained for 272 patients. Pathway‐level enrichment was evaluated using gene set variation analysis. scRNA‐seq datasets from mCRPC tissue biopsies and circulating tumor cells were used to investigate heterogeneity of adverse pathways. We identified five pathway clusters: (a) Immune response/WNT/TGF‐beta signaling, (b) AR signaling/luminal signatures, (c) mTOR signaling and glycolysis, (d) cell proliferation, and (e) neuroendocrine differentiation. Proliferation, AR signaling loss, and glycolysis/mTOR signaling were independently prognostic. Adverse prognostic pathway scores decreased on treatment with AR signaling inhibitors, but not at progression, suggesting failure to permanently target these pathways. scRNA‐seq datasets from mCRPC tissue biopsies and circulating tumor cells were used to investigate heterogeneity of adverse pathways. Our results suggest loss of AR signaling, high proliferation, and a glycolytic phenotype as adverse prognostic pathways in mCRPC that could be used in conjunction with clinical factors to prognosticate for treatment decisions.

## Graphical abstract

We aggregated a cohort of 1012 mCRPC tissue samples from 769 patients and investigated the association of gene expression‐based pathways with clinical outcomes. Loss of AR signaling, high proliferation, and a glycolytic phenotype were independently prognostic for poor outcomes, and an adverse transcriptional feature score incorporating these pathways added prognostic value to known clinical and genomic prognostic features.

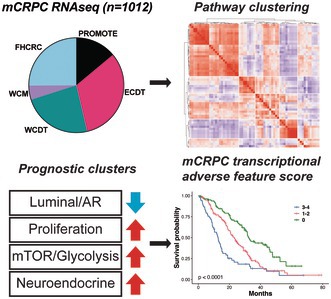

**Abbreviations**
ADT: androgen deprivation therapy
AR: androgen receptor
ASI: androgen signaling inhibitor
AVPC: aggressive variant prostate cancer
CTC: circulation tumor cell
DNA: deoxyribonucleic acid
ECDT: East Coast Dream Team
FHCRC: the Fred Hutchinson Cancer Research Center
GSEA: gene set enrichment analysis
GSVA: gene set variation analysis
HR: hazard ratio
mCRPC: metastatic castration‐resistant prostate cancer,
MSigDb: Molecular Signatures Database
NEPC: neuroendocrine prostate cancer
PROMOTE: Prostate Cancer Medically Optimized Genome‐Enhanced Therapy
PSA: prostate specific antigen
RNA: ribonucleic acid
scRNA‐seq: single‐cell RNA‐sequencing
SU2C/PCF: Stand Up 2 Cancer/Prostate Cancer Foundation
TPM: transcript per million
WCDT: West Coast Dream Team
WCM: Weill Cornell Medicine

## 1 Introduction

Prostate cancer is the most common malignancy in men, with most patients presenting with localized disease. The clinical behavior of the disease ranges from indolent to highly aggressive. Initial treatment is primarily with surgery or radiation, with or without androgen deprivation therapy (ADT). In patients who progress to metastatic disease, ADT forms the backbone of systemic therapy. However, many patients will eventually develop metastatic castration‐resistant prostate cancer (mCRPC), the lethal and end stage of the disease. Treatment typically involves AR signaling inhibitors (ASIs), though a wide range of other therapies are also available (e.g., chemotherapy, PSMA‐Lu177, Radium‐223, Sipuleucel‐T, and PARP inhibitors) making the treatment landscape increasingly complex. Much effort is directed towards establishing the sequencing and/or combinations and disease stages at which these therapies are most beneficial to patients, balancing treatment efficacy and toxicity and other side‐effects. Molecular phenotypic characterization of mCRPC could substantially aid this effort by selecting patients for a personalized treatment approach [1, 2].

DNA profiling studies have revealed that the transition to mCRPC is associated with frequent copy number amplification of *AR* and/or an upstream enhancer region in response to prolonged AR‐targeted therapy and that loss of multiple tumor suppressor genes (*TP53*, *PTEN*, *RB1*) are associated with development of neuroendocrine prostate cancer (NEPC), an aggressive androgen‐independent subtype with poor outcome [3, 4, 5, 6, 7]. However, DNA genomic alterations are not able to fully explain the heterogenous clinical behavior of mCRPC, and beyond alterations in homologous recombination repair genes (such as *BRCA2*) or microsatellite instability, genomic alterations are not currently used to inform the management of mCRPC patients.

Similarly, molecular studies of localized prostate cancer such as The Cancer Genome Atlas have revealed distinct subtypes characterized by key DNA alterations across a host of oncogenes and tumor suppressors [8]. However, gene expression studies of primary tumors have been more successful at defining clinically relevant subtypes and large efforts profiling thousands of samples have defined subtypes with prognostic implications, largely based on the luminal‐basal axis, proliferative pathways, and androgen signaling [9, 10, 11], and form the basis of several commercially available prognostic tests [12].

While tens of thousands of localized prostate cancer samples have been profiled for gene expression patterns, access to mCRPC tissue samples is more limited. Clinical sequencing efforts in mCRPC have identified prognostic subtypes analogous to those found in localized disease such as the luminal‐basal axis and the PCS1‐3 subtypes [9, 13]. Additional subtypes and prognostic groups have been identified primarily along the androgen signaling, proliferation, neuroendocrine axes [4], and immune infiltration [14] such as the MetA‐C classification [15, 16, 17], the five‐subtype schema proposed by Labrecque [18], pseudotime as marker of disease progression [19], and several of these subtypes are prognostic for survival in mCRPC [5, 20, 21].

However, our ability to comprehensively assess prognostic pathways has been limited by the challenge of obtaining metastatic biopsies for gene expression profiling, as well as heterogeneity in prior lines of therapy and timing of biopsy. Therefore, we aggregated 1012 mCRPC samples with RNA‐seq from eight studies representing five large clinical sequencing efforts and 769 unique patients into the largest combined mCRPC RNA‐seq cohort with batch effect corrected data. Using this cohort, we comprehensively investigated gene expression‐based pathways associated with clinical outcomes and sought to characterize association of adverse prognostic pathways with genomic and clinical features as well as intrapatient and intratumor heterogeneity.

## 2 Methods

**Fig. 1:**
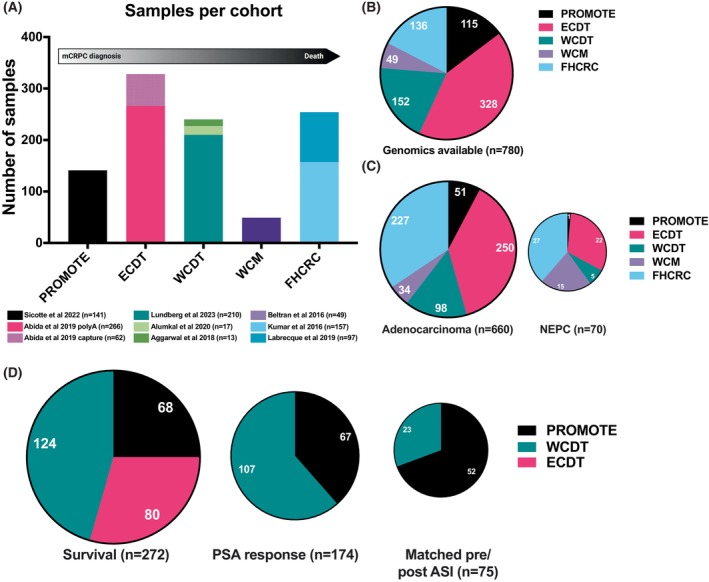
Composition of the combined mCRPC transcriptional profiling dataset. (A) mCRPC RNA‐sequencing data were collected from five large clinical cohorts comprising 1012 tissue samples from 769 patients across mCRPC disease stages, including the Prostate Cancer Medically Optimized Genome‐Enhanced Therapy (PROMOTE) study cohort, the Stand Up 2 Cancer/Prostate Cancer Foundation (SU2C/PCF) East Coast Dream Team (ECDT), the SU2C/PCF West Coast Dream Team (WCDT) mCRPC cohort, a neuroendocrine prostate cancer‐enriched cohort from Weill Cornell Medicine (WCM), and the Fred Hutchinson Cancer Research Center (FHCRC) autopsy cohort. (B) Distribution of samples from each cohort among all samples with DNA sequencing available. (C) Distribution of samples from each cohort among all adenocarcinoma and NEPC, (D) Distribution of samples from each cohort among the subset of patients with overall survival data, PSA response data, and matched pre‐/post‐ASI samples.

Clinical and processed RNA‐sequencing data were obtained from eight published studies from five sequencing efforts: the Fred Hutchinson Cancer Research Center (FHCRC) autopsy cohort (*n* = 254) [18, 22], a neuroendocrine prostate cancer‐enriched cohort from Weill Cornell Medicine (WCM) (*n* = 49) [4], the mCRPC cohort from the Stand Up 2 Cancer/Prostate Cancer Foundation (SU2C/PCF) East Coast Dream Team (ECDT) dataset (*n* = 328) [20, 23], the SU2C/PCF West Coast Dream Team (WCDT) mCRPC cohort (*n* = 240) [5, 7, 21, 24, 25, 26, 27], and the Prostate Cancer Medically Optimized Genome‐Enhanced Therapy (PROMOTE) study cohort (*n* = 141) [28] (Fig. 1A). Altogether, these samples represented 769 unique patients (Fig. S1A). Details of the study cohorts have been published elsewhere [4, 5, 7, 18, 20, 21, 22, 23, 24, 25, 26, 27, 28]. DNA alteration calls were available for 780 samples (Fig. 1B). Adenocarcinoma versus neuroendocrine prostate cancer (NEPC) status was available for 838 samples, as defined in each cohort (Fig. 1C). Clinical annotation was available for a subset of samples from the WCDT, ECDT, and PROMOTE cohorts (Fig. 1D), including overall survival (*n* = 272), PSA response (*n* = 174) (Fig. 1D), and prior ASI exposure status (*n* = 281) (Fig. S1B). Matched pre‐ and post‐ASI exposure samples were available for a subset of patients from the WCDT and PROMOTE cohorts (*n* = 75, Fig. 1D); we included only the pre‐ASI samples for all of the analyses with the exception of longitudinal pre‐/post‐ASI analysis in Fig. 6. DNA alteration calls were downloaded from cBioportal (www.cbioportal.org) for the FHCRC, WCM, and ECDT cohorts. The WCDT DNA alteration calls have been published previously [21]. Calls were used as provided for the ECDT, FHCRC and WCM cohorts. WCM copy number calls were classified as follows: below −1.1 for deep deletion, between −1.1 and −0.4 for deletion, between −0.4 and 0.3 for copy neutral, between 0.3 and 1.1 for gain, and above 1.1 for amplification. PROMOTE cohort DNA sequencing data were downloaded from dbGaP (phs001141) and variants were called on tumor samples using VardictJava [29] (v1.8.3) with default parameters followed by testing for strand bias with VardictJava ‘teststrandbias.R’ and then conversion to VCF using ‘var2vcf_valid.pl’ with default parameters. Variants were additionally called on matched germline samples in the same manner. Variants from the tumor samples were then filtered with these matched germline samples by removing any variant present in the corresponding matched germline. The final group of variants were then filtered using the following parameters: FILTER==PASS, depth (DP) >= 10 and variant depth (VD) >= 3, allele frequency (AF) >= 0.02. Finally, only nonsynonymous variants in protein coding regions were kept. Copy number alterations were called using CNVkit [30] (v0.9.10). For a normal reference, all germline samples were pooled together to create a single reference as recommended by the CNVkit protocol. Copy number calling was then performed on the tumor BAM files in batch mode while specifying the male reference flag (‐‐male‐reference). Target probe regions (‐‐targets) were taken from (https://github.com/AstraZeneca‐NGS/reference_data/tree/master/hg38/bed/Exome‐Agilent_V4.bed) to match the panel used for the targeted sequencing [31]. Copy number calls for each individual sample were then adjusted for purity and ploidy using the “call” command in CNVkit by setting the ‐‐purity and ‐‐ploidy flags with the known purity and ploidy data provided by the PROMOTE report [28]. For the PROMOTE cohort, samples with at least 15% tumor content were kept and default cutoffs for CNVkit were used.

We included genes with expression data in all samples. To adjust for batch effects between cohorts, we used the same approach we previously used for a subset of the FHCRC, WCM, WCDT, and ECDT cohorts [13]. Briefly, we first converted gene expression levels into per‐sample gene ranks, then corrected for batch effect using ComBat [32]. Sample‐level pathway enrichment was evaluated using gene set variation analysis (GSVA) [33] on the batch‐corrected gene expression dataset, utilizing the Molecular Signatures Database hallmark pathway gene sets [34] along with published gene signatures relevant to androgen signaling, prostate cancer subtyping, and luminal, basal, and neuroendocrine prostate cancer differentiation [4, 5, 15, 18, 21, 27, 35, 36, 37, 38, 39] (Table S1). Pathways were then clustered by Pearson's correlation coefficient across the combined cohort. Composite pathway scores were generated from the mean of individual pathway signatures that clustered together by Pearson's correlation coefficient and were associated with prognosis in univariate analysis. Immune content was quantified using CibersortX [40]. An analysis of additional pathways associated with prognosis in univariate cox proportional hazards models as described below was also performed using GSVA with the Wikipathways [41] and Pathway Interaction Database [42] gene signature sets from the MSigDB C2 canonical pathways gene sets collection, as well as prostate cancer subtype signatures from Tang et al. [43] and You et al. [9]. Finally, GSEA [44] was performed for the hallmark pathways and prostate cancer relevant signatures (Table S1) on all individual genes in the dataset ranked by hazard ratio in univariate cox proportional hazards models as described below. All gene expression analysis was completed in R version 4.2.2 (R Foundation for Statistical Computing).

Intrapatient heterogeneity analyses were performed where multiple site biopsy samples from the same patient were available. For single‐cell heterogeneity analyses, publicly available scRNA‐seq datasets from mCRPC tissue biopsies [45] and circulating tumor cells [46] were utilized. Some circulating tumor cell samples had very high hemoglobin (HBB) transcript levels, in some cases comprising the majority of sequenced reads, so *HBB* was removed from the count matrix prior to calculating TPM, to avoid biasing results. Sample‐level pathway enrichment was evaluated using gene set variation analysis (GSVA) of TPM gene expression values. Composite pathway scores were calculated as described above. Immune content was quantified from the sum of log_2_‐transformed expression values of CD45, CD14, CD16, CD11b, CD27, and CD3.

### 2.1 Statistical analysis

The primary clinical endpoint was overall survival, defined as time from first biopsy (if multiple biopsies per patient) to death from any cause or censored at time of last contact. Cox proportional hazards regression was used to assess the prognostic potential of each gene set. For each gene set, the continuous GSVA score was used, and the model was adjusted for biopsy site as a covariable and stratified for study cohort. *P*‐values were corrected for multiple testing using the Benjamini–Hochberg method, and adjusted *P*‐values of < 0.05 were considered significant. After calculation of composite pathway scores, a multivariable model including composite pathway scores and biopsy site, stratified for cohort was created.

Survival between groups was plotted using the Kaplan–Meier method, and a log‐rank test was used to compare differences in survival. To compare matched pre‐ and post‐ASI exposure gene set scores, a Wilcoxon signed‐rank test was used. To compare transcriptional adverse features scores between patients with and without PSA response defined as a ≥ 50% decrease in PSA, a Cochrane–Armitage test was used. All statistical analysis was completed in R version 4.2.2 (R Foundation for Statistical Computing, Vienna, Austria).

## 3 Results

### 3.1 Global assessment of pathways in mCRPC

**Fig. 2:**
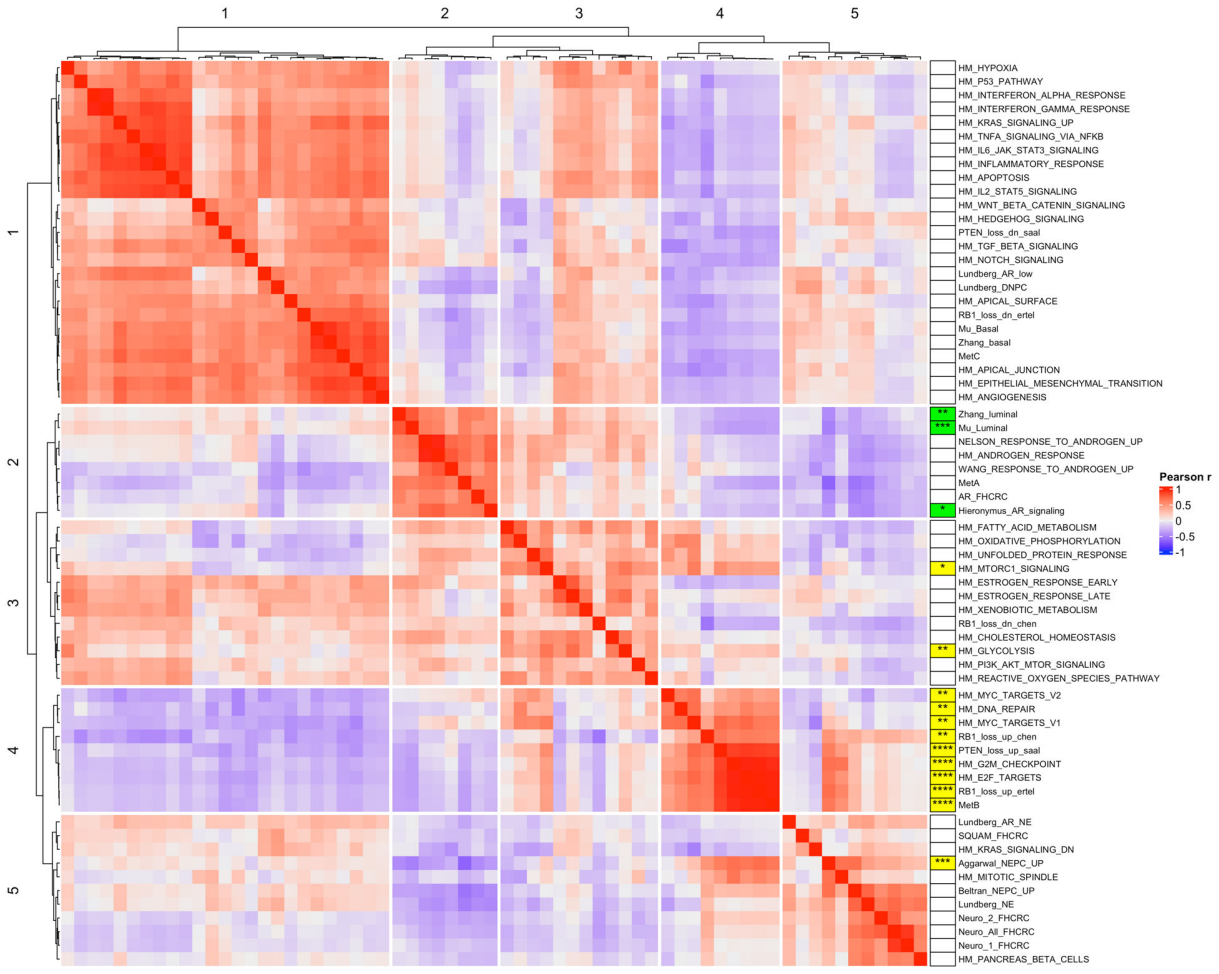
Correlation of hallmark and prostate associated pathways across the combined dataset and association with prognosis. Pearson's correlation of hallmark pathway and prostate‐related gene signatures across the combined cohort (*n* = 1012 samples) reveals five signaling clusters including (1) basal, mesenchymal and immune signatures, (2) luminal and androgen response signatures, (3) metabolic signatures, (4) proliferation and tumor suppressor loss signatures, and (5) neuroendocrine signatures. In univariate cox proportional hazards models of the survival subset (*n* = 272 patients) adjusted for biopsy site and stratified by cohort, signature scores associated with worse prognosis are labeled in yellow, while signature scores associated with improved prognosis are labeled in green, along with significance for each association (univariate cox proportional hazards model *P < 0.05, **P < 0.01, ***P < 0.001, ****P < 0.0001, corrected for multiple testing).

We first sought to globally characterize the relationships between the hallmark signaling and prostate‐cancer‐relevant pathways in our combined mCRPC dataset. To do this, we correlated the GSVA signature scores for all pathways with each other and performed hierarchical clustering, identifying five clusters of correlated pathways (Fig. 2). Cluster 1 included signatures associated with immune response, WNT and TGF beta signaling, epithelial‐mesenchymal transition, prostate cancer basal differentiation, and AR low/double negative prostate cancer subtypes. Cluster 2 comprised luminal and androgen signaling signatures, and Cluster 3 included metabolic signatures associated with fatty acid metabolism, oxidative phosphorylation, cholesterol homeostasis, mTOR pathway signaling and glycolysis. Cluster 4 comprised hallmark pathways associated with cell proliferation (G2M checkpoint, E2F targets, MYC targets), DNA repair and tumor suppressor loss, and the MetB signature associated with a low AR/high proliferation prostate cancer phenotype [15]. Finally, cluster 5 included the signatures associated with neuroendocrine prostate cancer differentiation, as well as the hallmark signature for pancreatic beta cells, a primary neuroendocrine cell type, and the hallmark mitotic spindle pathway.

### 3.2 Pathways associated with adverse clinical outcome

In parallel, we leveraged the samples annotated with overall survival outcomes to perform Cox regression of each pathway as a continuous variable, adjusted for biopsy site and stratified by sample cohort. After multiple testing correction, 15 pathways across pathway clusters 2–5 were significantly associated with overall survival (Fig. 2, Table S2). Pathways associated with favorable prognosis included signatures in the luminal/AR response cluster (cluster 2 or Luminal_AR), comprising both prostate luminal signatures [38, 39] and a classic androgen signaling signature [37]. Pathways associated with shorter survival included the hallmark pathways for mTORC1 signaling and glycolysis (cluster 3 or mTOR_glycolysis), all pathways in the proliferation/tumor suppressor loss cluster (cluster 4 or Proliferation), and the neuroendocrine prostate cancer signature developed by Aggarwal and colleagues [5] (cluster 5 or NEPC). We performed a similar secondary analysis of GSVA scores for MSigDB Wikipathways and Pathway Interaction Database C2 Canonical Pathways, which demonstrated the most significant association with adverse prognosis for pathways related to proliferation, cell cycle, and DNA repair, supporting the findings of our initial manually curated pathway analysis (Data S1). We also evaluated genesets associated with the prostate cancer subtype classifications reported by Tang et al. [43] and You et al. [9], finding that the Tang et al. neuroendocrine subtype signature as well as the You et al PCS1 highly proliferative luminal subtype signature were both significantly associated with poor prognosis in our dataset, again concordant with the pathways identified in our initial analysis (Data S1). Finally, GSEA of individual genes expression ranked by hazard ratio of association with survival in Cox regression using our manually curated pathway list again re‐identified the pathways associated with adverse prognosis in the GSVA analysis, further supporting our findings and identifying the specific genes driving these transcriptional axes by leading edge analysis (Data S1).

**Fig. 3:**
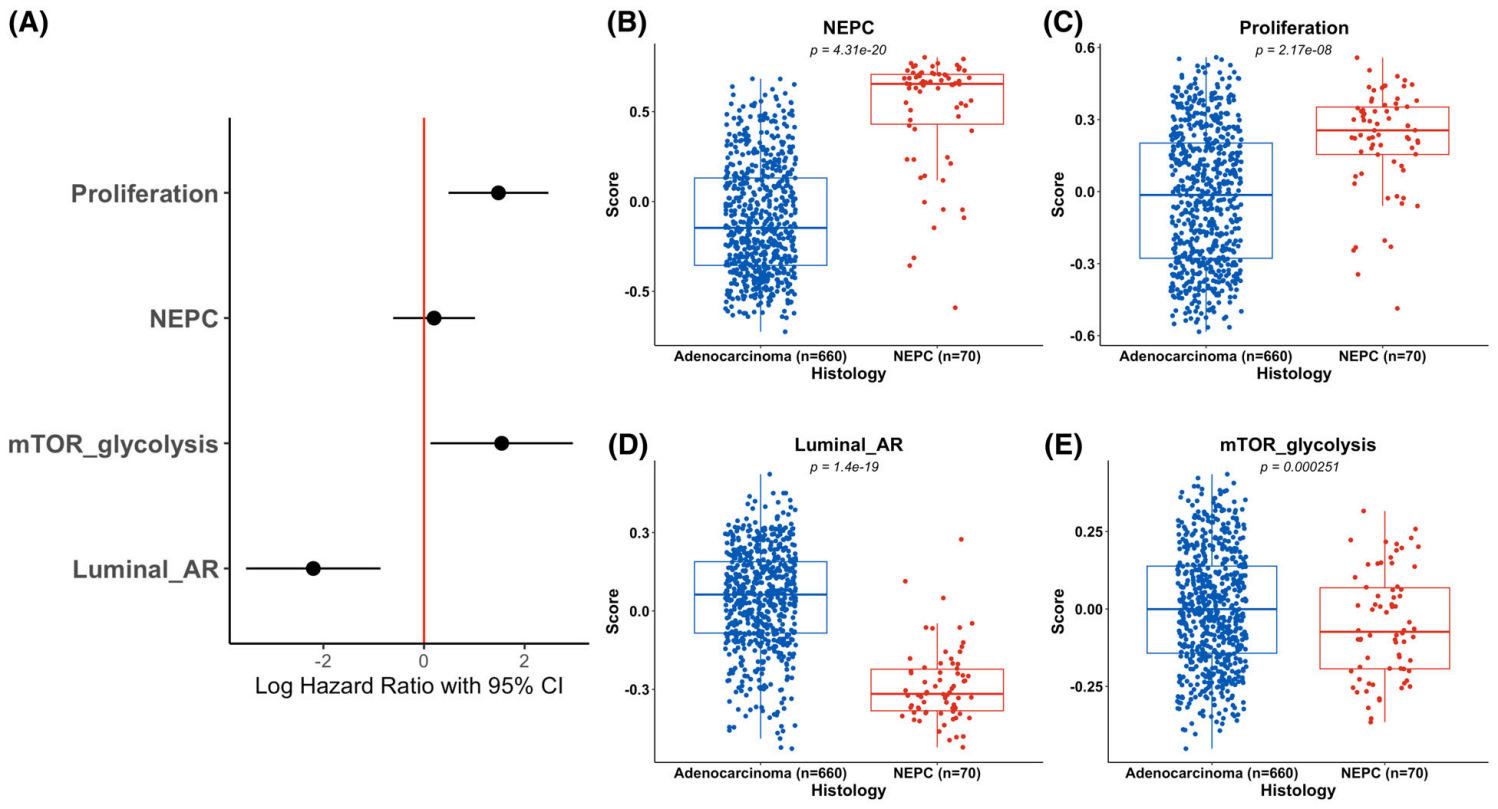
Prognostic signatures and histologic subtype. (A) Multivariate cox proportional hazards analysis in the survival subset (*n* = 272 patients) of combination prognostic signatures created from signatures associated with prognosis in each signature cluster including proliferation and tumor suppressor loss cluster (Proliferation), neuroendocrine cluster (NEPC), metabolism cluster (mTOR_glycolysis), and androgen response/luminal cluster (Luminal_AR), demonstrating independent prognostic value for the Proliferation (*P* = 0.003), mTOR_glycolysis (*P* = 0.032) and Luminal_AR (*P* = 0.001) cluster scores. Model is adjusted for biopsy site and cohort. The NEPC (B) and Proliferation (C) scores are increased in histologically defined NEPC (*n* = 70) compared to adenocarcinoma samples (*n* = 660), while the Luminal_AR (D) and mTOR_glycolysis (E) scores are decreased. Box and whiskers plots are shown with box representing interquartile range (IQR) split at median, and whiskers extending to minimum and maximum values within 1.5 × IQR; points represent all samples in each group.

We then performed a multivariable analysis to interrogate the independence of the four pathway clusters (clusters 2–5) associated with prognosis in the univariate analysis. We generated composite scores combining the pathways within each cluster that were individually associated with prognosis and constructed a multivariate model with each of these scores and biopsy site, stratified by sample cohort. In this model, the Proliferation (*P* = 0.003), mTOR_glycolysis (*P* = 0.032) and Luminal_AR (*P* = 0.001) composite pathway scores were independently prognostic (Fig. 3A). NEPC was not independently prognostic which is likely due to known association with proliferation. As expected, NEPC and proliferation scores were significantly higher in histologic NEPC samples compared to adenocarcinoma samples (Fig. 3B,C), while Luminal_AR scores were lower (Fig. 3D). mTOR_glycolysis scores (Fig. 3E) were also lower in NEPC compared to adenocarcinoma, suggesting that this may be an adverse prognostic feature specifically in the setting of adenocarcinoma.

Taken together, this analysis singles out key transcriptional axes independently associated with adverse clinical outcomes in mCRPC: increased cell proliferation, increased mTOR signaling and glycolysis, and decreased androgen signaling/luminal differentiation. In our univariate analysis, neuroendocrine differentiation was also associated with adverse prognosis as expected, but likely due to the relationship between neuroendocrine differentiation, increased proliferation and decreased androgen signaling, was not found to be independently prognostic in multivariate analysis, unlike the other composite pathways.

### 3.3 Expression patterns are associated with metastatic site and genomic alterations

**Fig. 4:**
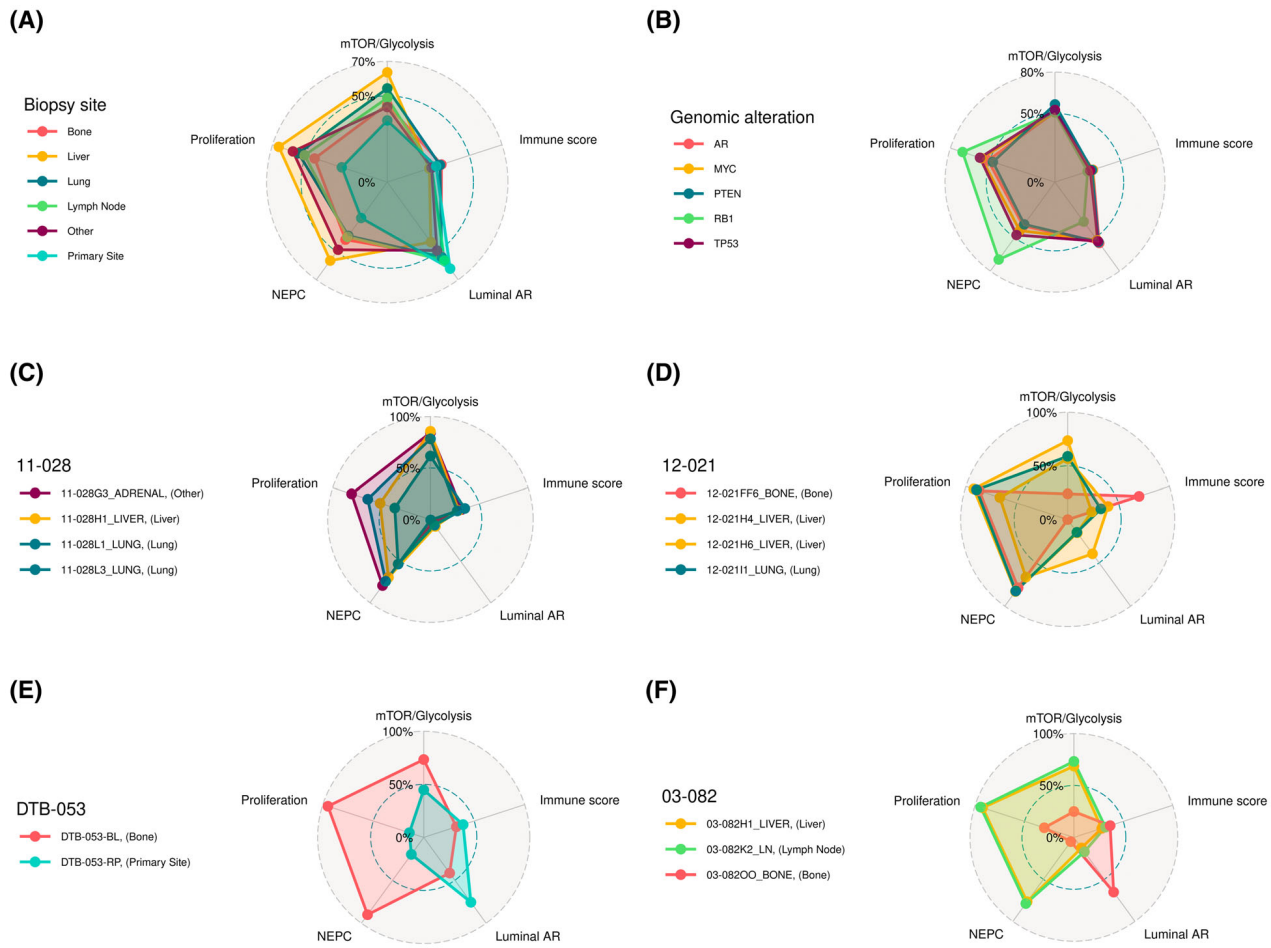
Expression signatures of adverse prognosis pathways per metastatic site, genomic alteration, and examples of intrapatient homogeneity and heterogeneity. (A) Association of expression signatures with metastatic site (Primary site *n* = 30, Bone *n* = 321, Liver *n* = 124, Lung *n* = 46, Lymph node *n* = 316, Other *n* = 87) (B) Association of expression signatures with genomic alterations (AR mutation or amplification *n* = 582, MYC amplification *n* = 498, PTEN 2‐copy loss *n* = 224, RB1 2‐copy loss *n* = 108, TP53 2‐copy loss *n* = 187) (B). Expression signatures from different tissue sites in a single patient can be concordant (C, D) or discordant (E, F). For this analysis, pathway scores were rescaled from 0% to 100% over all 1012 samples. For A and B, median pathway score was taken across all samples from a metastatic site or genomic alteration. If a sample had more than one genomic alteration the sample was included in more than one group.

We next sought to understand the relationship between the adverse transcriptional features and established clinicogenomic prognostic factors. First, we evaluated the relationship of these transcriptional features with metastatic site and immune infiltration. A radar plot of median composite pathway scores by biopsy site (Fig. 4A) demonstrates that all metastatic sites had higher mTOR_glycolysis, proliferation and NEPC scores than primary site biopsies, while primary sites had the highest luminal scores. This was most pronounced for liver metastases (known to be a poor prognosis metastatic site) [47, 48], while conversely bone metastases had the lowest proliferation, NEPC and mTOR_glycolysis composite scores across metastatic sites. We then evaluated the relationship between the adverse transcriptional features and DNA alterations associated with poor prognosis, including *AR*, *RB1*, *PTEN*, *TP53* and *MYC* alterations. A radar plot of median composite pathway scores by genomic alteration status (Fig. 4B) demonstrates that tumors with *RB1* loss had notably higher proliferation and NEPC scores and lower luminal scores than other genomic alterations, concordant with the established role for *RB1* loss in aggressive disease and transition to neuroendocrine differentiation [6]. Indeed, a univariate cox regression analysis for each DNA alteration in the survival dataset adjusted for biopsy site and stratified by cohort demonstrated an association between two copy *RB1* loss and worse survival (Table S3). However, *AR*, *PTEN*, *TP53*, and *MYC* DNA alterations did not have a significant association with survival in this dataset, concordant with prior analyses of both the WCDT [27] and ECDT [20] cohorts, which comprise the majority of the samples with survival outcomes in our combined cohort. Finally, we leveraged the subset of 281 samples in the ECDT and WCDT cohorts annotated with prior ASI exposure to understand the relationship between ASI exposure and our transcriptional features. While luminal_AR composite scores were as expected lower in samples with prior ASI exposure compared with ASI naïve samples, none of the other scores were associated with ASI exposure status (Fig. S2).

### 3.4 Intrapatient heterogeneity of transcriptional adverse features

While seeding can occur across metastatic sites, most metastatic prostate cancers are considered to have a dominant clone selected through evolutionary bottlenecks and corresponding to limited intrapatient genomic heterogeniety [49]. Autopsy studies have largely confirmed intrapatient subtype homogeneity at the transcriptional level, although with some patients exhibiting clear heterogeneity [18, 50]. We here sought to characterize intrapatient heterogeneity of the composite adverse pathways. To do this, we leveraged cases with synchronous multisite biopsies, primarily from the FHCRC autopsy cohort, in which considerable homogeneity has been reported previously with a minority of patients exhibiting subtype heterogeneity [18, 22]. Overall, we observed homogeneity in composite pathway scores between tissue biopsy sites in the same patient (Fig. 4C,D), but there was variability in the degree of heterogeneity. In some patients, only small differences are seen between sites (Fig. 4C), whereas there was more diversity in other patients (Fig. 4D). One patient demonstrated a significant shift between primary site and bone metastasis (Fig. 4E), with a decrease in luminal score and marked increase in proliferation, mTOR_glycolysis and NEPC scores. Another patient had liver and lymph node metastases with concordant profiles, with a discordant bone metastasis demonstrating lower proliferation, mTOR_glycolysis and NEPC scores and a higher luminal score (Fig. 4F).

### 3.5 Intratumor heterogeneity of transcriptional adverse features

**Fig. 5:**
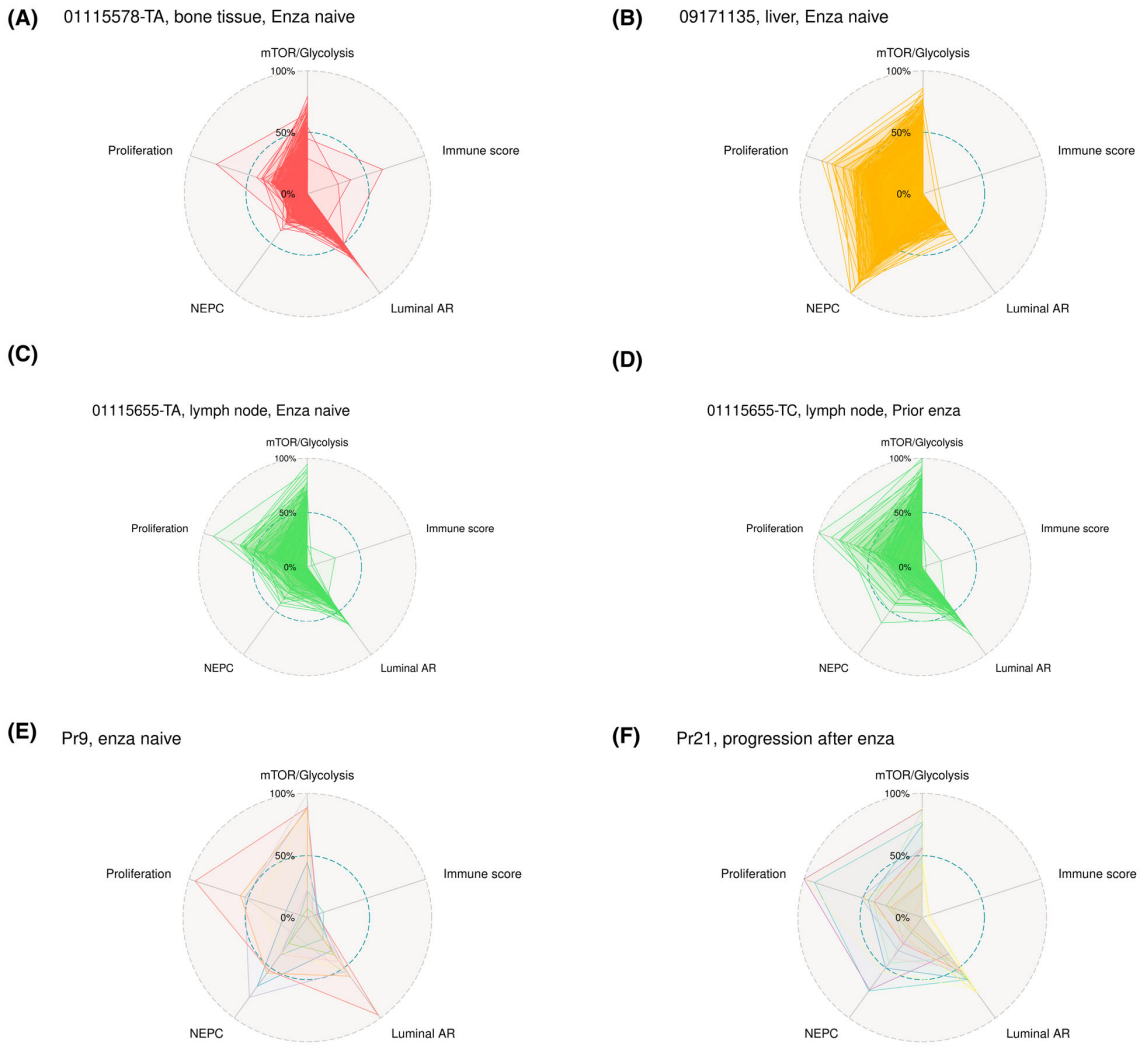
Adverse prognosis pathways show homogeneity and heterogeneity at the single‐cell level. Single‐cell RNA‐seq from an adenocarcinoma bone metastatic tumor (*n* = 338 single cells) (A) and a liver neuroendocrine metastatic tumor (*n* = 166 single cells) (B) show distinct patient specific patterns. Single‐cell RNA‐seq from a lymph node sample before (*n* = 159 single cells) (C) and after (*n* = 265 single cells) (D) enzalutamide show more cells with high proliferation and NEPC scores after enzalutamide. Considerable interpatient heterogeneity of adverse pathways was observed in CTCs both before (*n* = 9 CTCs) (E) and after (*n* = 12 CTCs) (F) enzalutamide. Pathway scores were rescaled from 0% to 100% over all prostate cancer cells per study (tissue single‐cell or CTC single‐cell RNA‐seq).

To understand whether the intrapatient heterogeneity in these composite adverse pathways extends to the intratumoral level, we utilized publicly available single‐cell RNA‐seq data from mCRPC tissue biopsies and circulating tumor cells to evaluate the composite pathway scores at the single‐cell level [45, 46]. Across tissue biopsy sites, most of the single cells within a given metastatic biopsy had similar profiles, although with some outlier cells (Fig. S3). Single‐cell sequencing of bone metastatic adenocarcinoma (Fig. 5A) and liver neuroendocrine tumor (Fig. 5B) demonstrated distinct profiles consistent with the bulk sequencing results, with higher proliferation and NEPC scores in the liver neuroendocrine tumor cells as expected. Within a patient, single‐cell sequencing of a lymph node biopsy before and after enzalutamide exposure (Fig. 5C,D) demonstrated more cells with high proliferation and neuroendocrine scores after enzalutamide treatment. In contrast to single‐cell sequencing of tissue biopsies, single‐cell sequencing of prostate circulating tumor cells demonstrated marked heterogeneity in composite pathway scores, as indicated by the original authors and subsequent reports investigating the PCS1‐3 subtypes [9, 46], (Fig. S4), which may reflect shedding from multiple metastatic sites into the circulation in the setting of intrapatient heterogeneity. Interestingly, this single‐cell level intrapatient heterogeneity was seen in both enzalutamide naïve (Fig. 5E) and postenzalutamide samples (Fig. 5F), though these were not matched samples from the same patients.

### 3.6 Dynamic changes in adverse pathways after ASI treatment

**Fig. 6:**
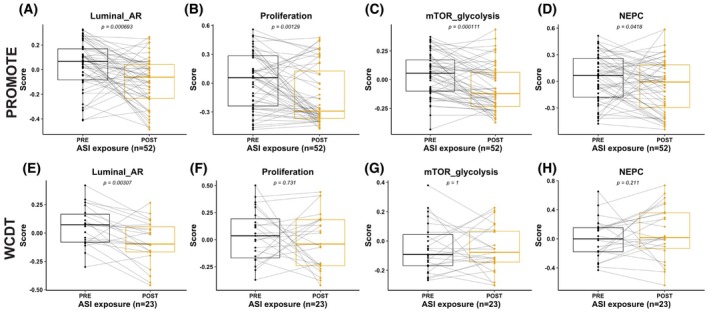
Prognostic signatures are decreased on treatment with ASI. Pre‐ versus early on‐treatment biopsies in the PROMOTE cohort (*n* = 52) demonstrate a significant decrease in Luminal_AR, mTOR_glycolysis, and Proliferation scores (A–C) on ASI treatment, while no significant change is seen in NEPC signatures (D). In contrast, only the Luminal_AR score is significantly decreased between matched pretreatment and post‐ASI progression biopsies in the WCDT cohort (*n* = 23) (E–H). Wilcoxon signed‐rank test is used to compare groups. Box and whiskers plots are shown with box representing interquartile range (IQR) split at median, and whiskers extending to minimum and maximum values within 1.5 × IQR; points represent all samples in each group and lines connect samples from the same patient.

Virtually all patients with mCRPC are now treated with ASIs early in the course of their disease. To further understand how ASI treatment impacts the composite adverse pathways, we took advantage of matched samples in the PROMOTE cohort collected prior to starting ASI and after 12 weeks on treatment [28]. Sicotte et al. identified differences in expression of genes involved in cell cycle, MYC target, DNA repair, and Wnt signaling in the on‐treatment biopsies of ASI non‐responders in this cohort, and a higher rate of mutations activating the PI3K/AKT/mTOR pathway [28]. We observed a significant decrease in luminal_AR, proliferation, and mTOR_glycolysis scores in the on‐treatment samples regardless of clinical response status (Fig. 6A–C), but no significant difference in NEPC scores (Fig. 6D). The decline in all three scores on treatment may reflect on‐target activity of ASI therapy rather than permanent modulation of the biology of the tumors.

To better understand whether on‐treatment ASI effects on the adverse features scores persist at time of ASI progression, we utilized matched samples in the WCDT cohort taken pre‐ASI and at the time of progression [25]. In contrast to the on‐treatment samples in the PROMOTE cohort, comparison between matched pretreatment and post‐ASI resistance samples in the WCDT cohort revealed a decrease in luminal_AR score but no other significant differences in the adverse feature scores (Fig. 6E–H). This is consistent with our findings in the larger cohort of unmatched ASI naïve versus ASI exposed samples (Fig. S2) as well as the prior analysis of a subset of the WCDT cohort by Westbrook et al. which did not find any consistent transcriptional shifts in the post‐treatment samples, with significant between‐patient heterogeneity in transcriptional phenotypes including AR signaling and evidence of NEPC differentiation, except in three patients that converted to a NEPC phenotype [25]. In combination with our analysis of the early on treatment samples from the PROMOTE cohort, this data suggests that one mechanism of acquired ASI resistance is failure of ASI to permanently suppress these composite adverse pathways.

### 3.7 Cumulative effects of multiple adverse pathway features

**Fig. 7:**
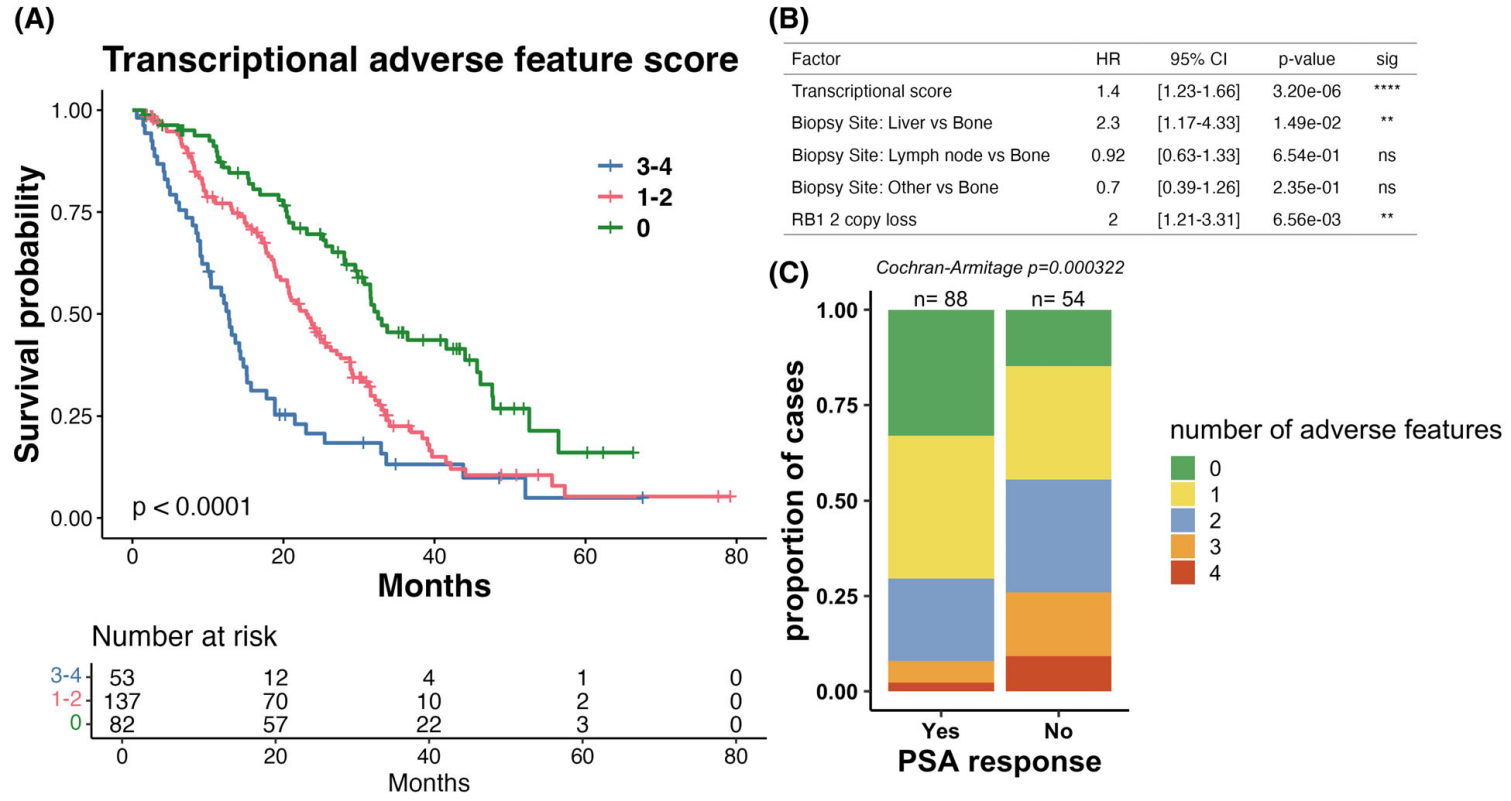
A combined transcriptional adverse feature score is prognostic for overall survival. (A) Patients with 3–4 adverse transcriptional features defined as (1) Luminal_AR score in the lowest tertile (2) mTOR_glycolysis score in the highest tertile, (3) Proliferation score in the highest tertile and (4) NEPC score in the highest tertile have significantly shorter survival than patients with 1–2 adverse features, while patients with no adverse features have improved survival (log‐rank *P* < 0.0001). (B) transcriptional adverse feature score is prognostic of survival independent of tumor site and genomic RB1 loss status in a multivariate cox proportional hazards model. (C) In patients who received an ASI postbiopsy and for whom PSA50 response was available (*n* = 142), number of transcriptional adverse features is significantly associated with likelihood of PSA response. Cochran‐Armitage test for trend is used to compare groups.

Finally, we sought to understand the combined effects of alterations in multiple composite adverse pathways, and whether the presence of adverse transcriptional feature could identify patients with more aggressive disease. To accomplish this, we defined categorical transcriptional adverse features as the top tertile for the composite pathway scores associated with poor outcome (proliferation, mTOR_glycolysis, and NEPC) or the bottom tertile for the composite pathway score associated with favorable outcome (luminal_AR). Outcomes for patients with zero adverse features were the best, followed by those with 1–2 features, with patients that had 3–4 adverse features at baseline as the worst subgroup (Fig. 7A). Importantly, this adverse transcriptional feature score was prognostic independent of both metastatic site and *RB1* DNA loss, the only DNA alteration associated with prognosis in this cohort (Fig. 7B). It also remained prognostic in each individual cohort with survival data (Fig. S5A–C). Finally, in the patients in the cohort who received an ASI postbiopsy and for whom PSA50 response was available the number of transcriptional adverse features at baseline was significantly correlated with likelihood of PSA response to ASI (Fig. 7C). Taken together, these gene expression pathways stratify mCRPC patient outcomes beyond known clinicogenomic prognostic factors and may identify patient populations in which escalated or de‐escalated therapy approaches could be evaluated.

## 4 Discussion

Herein, we have combined eight studies with a total of 1012 mCRPC samples from 769 patients representing five large clinical sequencing efforts to globally investigate the transcriptional landscape associated with aggressive disease in mCRPC. This allowed us to identify four crucial transcriptional axes associated with poor survival: low luminal/androgen receptor signaling, high cell proliferation, high mTOR signaling/glycolysis, and neuroendocrine differentiation. This builds on previous preclinical, and in some cases, clinical data implicating these pathways individually in prostate cancer progression and treatment resistance. However, by leveraging a large cohort of samples for which both transcriptional profiling and clinical outcomes are available, we are able to perform an integrated survival analysis demonstrating that three of these four axes are independently prognostic of overall survival, and that the prognostic impact of these axes is cumulative, such that a transcriptional adverse feature score combining these four axes can identify patients with aggressive disease independent of known clinicogenomic prognostic factors.

While most metastatic prostate cancers remain dependent on androgen signaling at the time of progression to castrate resistance, molecular subtypes of mCRPC with low androgen signaling activity have been described [18] after exposure to ASI therapy. This includes a subset of mCRPC patients whose tumors undergo transformation to an androgen‐independent neuroendocrine phenotype, a highly aggressive form of ASI resistance [4, 5, 18]. Low androgen response signature scores have also been associated with ASI (enzalutamide) resistance in a subset of the WCDT cohort [24]. Conversely, a prior transcriptional analysis of a subset of the ECDT and WCDT cohorts demonstrated an association between higher luminal transcriptional phenotype, androgen signaling scores, and favorable prognosis, particularly in the setting of ASI therapy [13]. In our multivariable analyses, it is striking that loss of AR signaling/luminal phenotype is a stronger prognostic feature than gain of an NEPC phenotype. This is in line with the hypothesis that a general de‐differentiation and lineage plasticity in prostate cancer is more prevalent than the specific case of clear NEPC differentiation [51]. Additionally, despite sustained AR signaling in mCRPC through gene amplification and rearrangements, the functional targets of AR undergoes extensive reprogramming away from a luminal program [52]. This is also line with the study by Bolis et al. [19], in which loss of androgen response and increase in proliferation and E2F target expression were the pathways most strongly correlated with pseudotime for disease progression, although this study did not evaluate the association with survival.

Our results reinforce high cell proliferation as strongly prognostic in mCRPC, perhaps the most well‐known indicator of aggressive cancer across disease sites. This cluster included signatures of loss of function of the tumor suppressors RB1 and PTEN, which have been associated with aggressive disease across many solid tumors including prostate cancer. While the proliferation signatures are suppressed in biopsies from patients on treatment with ASI therapy, this suppression is no longer observed at the time of progression. Single‐cell analyses also indicated an increase in the number of tumor cells with high proliferation signatures and NEPC scores after ASI treatment. RB1, TP53 and PTEN loss have been associated with lineage plasticity and transition to a neuroendocrine phenotype [3, 4, 5], however neuroendocrine signatures clustered separately but distinct from the cell proliferation/tumor suppressor loss cluster in our combined cohort. Since RB1, TP53, and PTEN loss are enriched in NEPC but also present in a subset of prostate adenocarcinoma [23], this observation highlights that while RB1 and PTEN loss more frequent in cancer with neuroendocrine differentiation, loss of these tumor suppressors is associated with aggressive mCRPC even in the absence of neuroendocrine differentiation. We also find that the transcriptional adverse features described in this manuscript are prognostic independent of genomic *TP53, RB1 and PTEN* alterations, and notably only *RB1* two copy loss had any significant association with survival in our combined cohort, highlighting the additional prognostic information that can be extracted from transcriptional features beyond genomic alteration status.

The metabolic shift to aerobic glycolysis (the “Warburg” effect) is a an established phenomenon across many solid tumor types [53], which to the best of our knowledge has not previously been associated with outcomes when measured in tissue biopsies. However, normal prostate cells already have an altered glucose metabolism, and a “dual switch” is proposed in which localized prostate cancer first increases oxidative phosphorylation and then a glycolytic switch occurs in late‐stage disease [54]. Across tumor types, this shift is commonly driven by activation of the PI3K‐AKT–mTOR pathway, a key cell growth and survival pathway [55] that links cell proliferation and survival with glucose metabolism [56]. The PI3K pathway is frequently activated in metastatic prostate cancer, most commonly through deletion or mutation of key negative regulator PTEN, which is altered in up to 50% of metastatic PCa [20, 23, 57, 58] and associated with poor prognosis [59]. The shift to aerobic glycolysis has recently has been linked to aggressive variant prostate cancer in prostate PDX models [60], and has also been shown to contribute to an immune suppressed tumor microenvironment in mouse models of PTEN deficient prostate cancer through modulation of tumor associated macrophage activity [61]. In our combined mCRPC cohort, hallmark pathways for glycolysis, mTORC1 signaling, PI3K/AKT signaling and additional metabolic pathways including fatty acid metabolism and oxidative phosphorylation clustered together. Additionally, high glycolysis and mTOR signaling pathway activity was associated with poor survival. Interestingly, high glycolytic activity in mCRPC has recently been suggested as both a prognostic imaging biomarker [62], and a therapeutic target [63], reinforcing the clinical relevance. Intriguingly, these metabolic pathways did not cluster with signatures of PTEN loss of function, and the mTOR_glycolysis combined pathway score was not different between PTEN altered and non‐altered tumors, suggesting that this metabolic dysregulation and associated aggressive disease biology may be driven by other mechanisms in addition to PTEN loss. Indeed, genomic alterations in multiple PI3K pathway genes besides PTEN have been identified in advanced prostate cancer [28, 64], and cross‐talk between the PI3K‐AKT–mTOR pathway and other pathways including AR signaling [65], Wnt [66], and Ras/MAPK [67, 68] pathways can converge on mTOR pathway activation in the absence of PI3K pathway genomic alterations altogether. Intriguingly, the mTOR/glycolysis combined pathway score was significantly lower in NEPC compared to adenocarcinoma, suggesting that this metabolic dysregulation may represent a distinct mechanism of aggressive disease from the transition to the androgen‐independent NEPC phenotype.

Despite aggregating a cohort of over 1000 mCRPC tissue samples, our study has limitations. First, we primarily analyze bulk RNA‐seq data, which have inherent limitations of adjacent normal tissue contamination (though we adjust by biopsy site) and cell type heterogeneity. While we do analyze available single‐cell data, the availability of large such datasets with clinical outcomes is still limited, and we are eagerly looking forward to future studies and sequencing efforts to explore this aspect in more depth. Another limitation of this study is the clinical heterogeneity between the cohorts and the potential lead time bias and prior treatment history that we could not fully account for. Thus, the association of these adverse pathways with decreased survival may indicate later disease stages rather than a distinct biological entity at baseline. Indeed, the pseudotime analysis by Bolis et al., suggest that increased proliferation and loss of AR signaling are strongly correlated with disease progression.

## 5 Conclusion

In conclusion, by aggregating a cohort of RNA‐seq data from 1012 mCRPC tissue biopsies our results reinforce and single out the prognostic strength of loss of AR signaling, high proliferation, NEPC differentiation, and a metabolic shift to a glycolytic phenotype in mCRPC. As RNA‐seq data becomes more readily available as a part of clinical sequencing panels, gene expression profiles can be used in conjunction with clinical factors to prognosticate and make treatment decisions. However, our data suggest that inter‐tumoral, and especially intra‐tumoral heterogeneity will continue to be a potential confounder of tissue sequencing. Emerging liquid biopsy technologies may provide a solution.

## Conflict of interest

KTH has a family member who is an employee of Epic Systems. YS reports employment at Tempus with restricted stock units. MB has a family member who is an employee of Luminex. SGZ reports unrelated patents licensed to Veracyte, and that a family member is an employee of Artera and holds stock in Exact Sciences. SMD reports consulting relationships with BMS, Oncternal therapeutics, Janssen R&D/J&J and a grant from Pfizer/Astellas/Medivation (the grant was submitted to Medivation, ultimately funded by Astellas and then moved to Pfizer). EJS reports honoraria from Janssen for serving on Advisory Board and honoraria and stock options from Fortis Therapeutics. MNS reports institutional research support from Novartis. MS reports speaker fees from Astellas and consulting fees for serving on Advisory Board from Veracyte/Adelphi Targis.

## Author contributions

MNS, SGZ, and MS conceived and designed the project. MNS, MK, RY, SMD, EJS, RA, DAQ, SGZ, and MS acquired the data. MNS, ErF, EmF, KTH, MLB, AS, YS, SGZ, and MS analyzed the data. All authors helped interpret the data. MNS, SGZ, and MS drafted the paper, all authors approved the final manuscript.

## Peer review

The peer review history for this article is available at https://www.webofscience.com/api/gateway/wos/peer‐review/10.1002/1878‐0261.70001.

## Supporting information


**Data S1.** Univariate Cox model results for MSigDB Wikipathways, Pathway Interaction Database C2 Canonical Pathways. Genesets associated with the prostate cancer subtype classifications reported by Tang et al. [43] and You et al. [9], and GSEA results of expression of individual genes ranked by hazard ratio of association with survival in Cox regression.

**Fig. S1.** Additional clinical parameters in the combined cohort. (A) Unique patients in each cohort/publication (*n* = 769). (B) Prior ASI exposure status was available for a subset of samples (*n* = 281) in the ECDT and WCDT cohorts.

**Fig. S2.** Association between prior ASI exposure and prognostic signatures. In the subset of samples from the ECDT and WCDT cohorts annotated for prior ASI exposure, the luminal_AR signature was decreased in samples with previous ASI (*n* = 184) compared to ASI naïve samples (*n* = 97), but the other signatures were not significantly different between groups.

**Fig. S3.** Adverse prognosis pathway scores in tissue single‐cell RNA‐seq data. Pathway scores were rescaled from 0% to 100% over all prostate cancer cells in the dataset. Single cells sequenced per sample: 01971144 *n* = 437, 01971146 *n* = 4, 01115149‐TC *n* = 261, 01115578‐TA *n* = 301, 01115666‐TA *n* = 101, 01115681 *n* = 26, 01115655‐TA *n* = 159, 01115655‐TC *n* = 265, 01115680 *n* = 97, 09171111 *n* = 10, 09171123 *n* = 97, 09171135 *n* = 166.

**Fig. S4.** Adverse prognosis pathway scores in CTCs from single‐cell RNA‐seq data. Pathway scores were rescaled from 0% to 100% over all prostate cancer CTCs in the dataset. Single CTCs sequenced per sample: Pr3 *n* = 1, Pr6 *n* = 3, Pr9 *n* = 9, Pr10 *n* = 1, Pr11 *n* = 11, Pr14 *n* = 11, Pr15 *n* = 1, Pr17 *n* = 4, Pr18 *n* = 9, Pr19 *n* = 4, Pr20 *n* = 1, Pr21 *n* = 12, Pr22 *n* = 10. Radar plots are colored by hemoglobin (HBB) expression which was variable between single CTC samples.

**Fig. S5.** Combined transcriptional adverse feature score in each mCRPC cohort. Higher transcriptional adverse feature score is associated with worse overall survival across all three separate survival cohorts including (A) the PROMOTE trial cohort, (B) the ECDT cohort and (C) the WCDT cohort.

**Table S1.** Prostate gene signatures.
**Table S2.** Univariate analysis associating each signaling pathway with survival. Univariate cox proportional hazards model hazard ratios and unadjusted and adjusted *P*‐values for association of each signaling pathway with overall survival in the survival subset (*n* = 272).
**Table S3.** Univariate analysis associating each genomic alteration with survival. Univariate cox proportional hazards model hazard ratios and unadjusted and adjusted *P*‐values for association of genomic alterations in *AR, MYC, PTEN, RB1* and *TP53* with overall survival in the survival subset (*n* = 272).

## Data Availability

Processed RNA and DNA sequencing data was downloaded from cBioportal (www.cbioportal.org) for the ECDT, FHCRC, and WCM cohorts. Further RNA‐sequencing data for the FHCRC cohort was downloaded from Gene Expression Omnibus with accession number GSE126078. WCDT RNA and DNA sequencing were previously published in Lundberg et al. [21], Westbrook et al. [25] and Alumkal et al. [24]. PROMOTE RNA‐sequencing was previously published in Sicotte et al. [28] and DNA sequencing data was downloaded from dbGAP with accession number phs001141.v2.p1.
