# Supplementary figures and images for: Adverse prognosis gene expression patterns in metastatic castration‐resistant prostate cancer
Source: Mol Oncol. 2025 Feb 22;19(8):2348–65. doi: 10.1002/1878-0261.70001 (PMC12330944; doi:10.1002/1878-0261.70001)

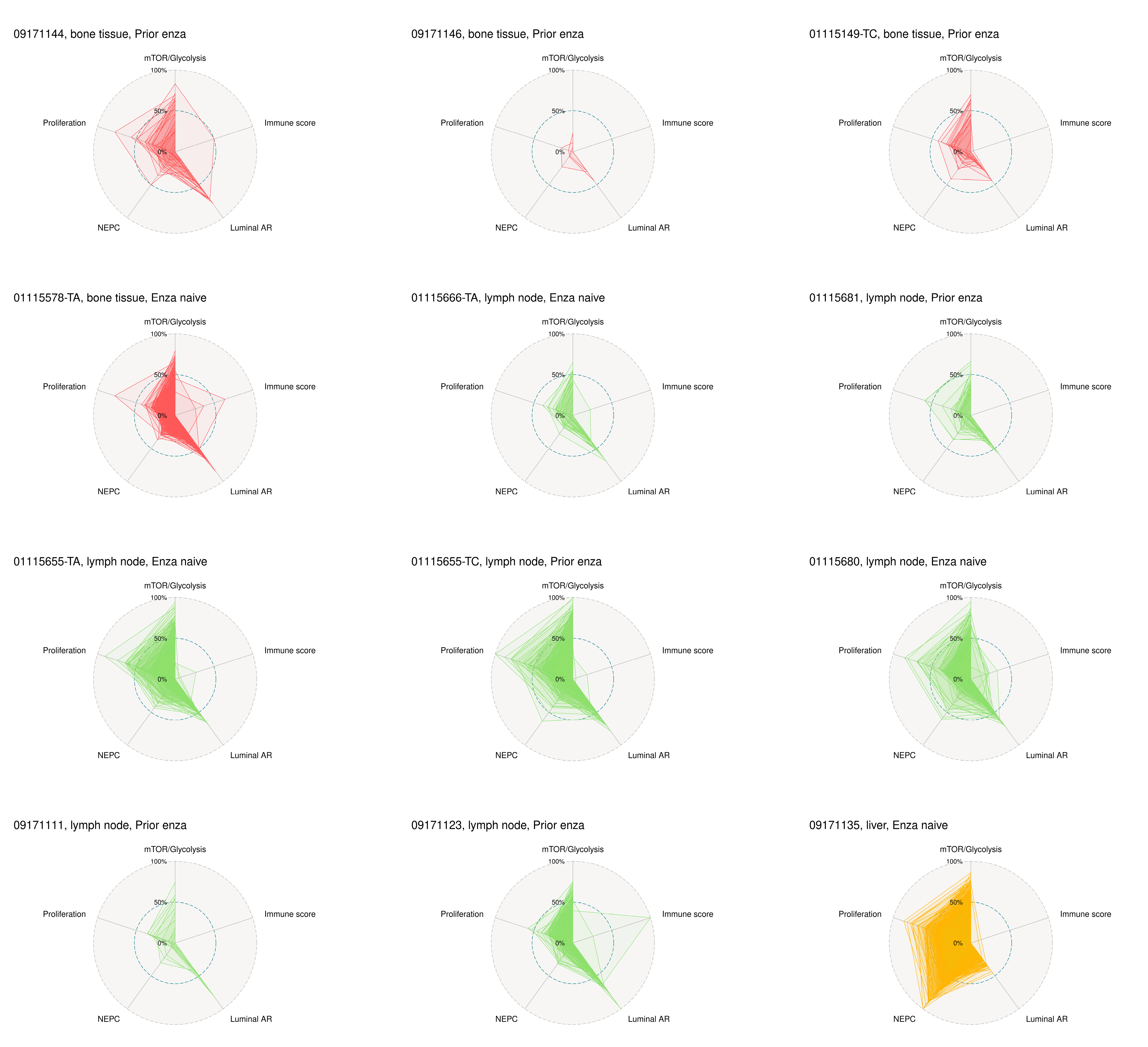

Supplement: Supplementary file 4 — Fig. S3. Adverse prognosis pathway scores in tissue single‐cell RNA‐seq data. Pathway scores were rescaled from 0% to 100% over all prostate cancer cells in the dataset. Single cells sequenced per sample: 01971144 n = 437, 01971146 n = 4, 01115149‐TC n = 261, 01115578‐TA n = 301, 01115666‐TA n = 101, 01115681 n = 26, 01115655‐TA n = 159, 01115655‐TC n = 265, 01115680 n = 97, 09171111 n = 10, 09171123 n = 97, 09171135 n = 166. [file MOL2-19-2348-s002.png]

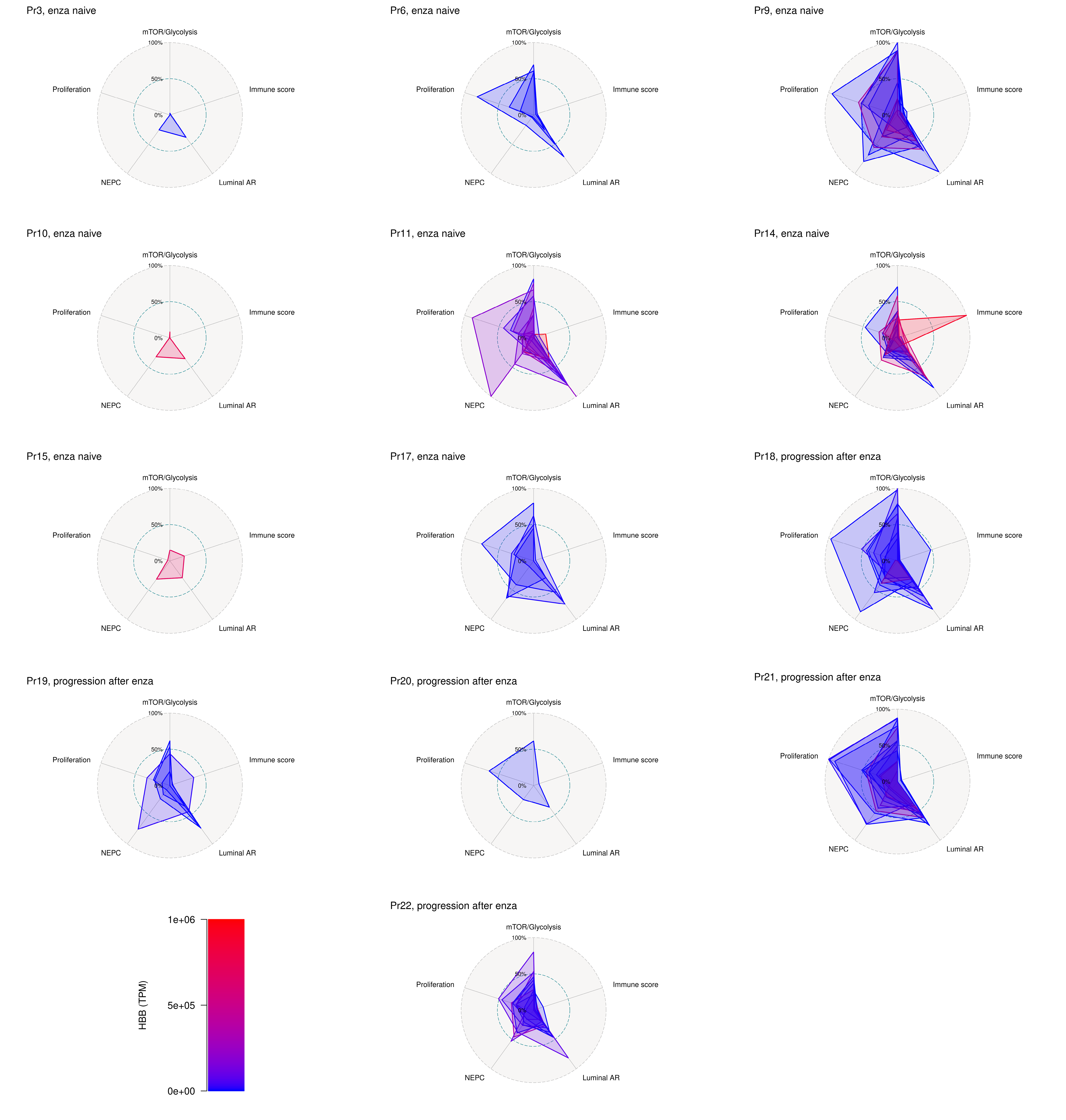

Supplement: Supplementary file 5 — Fig. S4. Adverse prognosis pathway scores in CTCs from single‐cell RNA‐seq data. Pathway scores were rescaled from 0% to 100% over all prostate cancer CTCs in the dataset. Single CTCs sequenced per sample: Pr3 n = 1, Pr6 n = 3, Pr9 n = 9, Pr10 n = 1, Pr11 n = 11, Pr14 n = 11, Pr15 n = 1, Pr17 n = 4, Pr18 n = 9, Pr19 n = 4, Pr20 n = 1, Pr21 n = 12, Pr22 n = 10. Radar plots are colored by hemoglobin (HBB) expression which was variable between single CTC samples. [file MOL2-19-2348-s004.png]
