# Supplementary material for: Adverse prognosis gene expression patterns in metastatic castration‐resistant prostate cancer
Source: Mol Oncol. 2025 Feb 22;19(8):2348–65. doi: 10.1002/1878-0261.70001 (PMC12330944; doi:10.1002/1878-0261.70001)
Supplement: Supplementary file 7 — Table S1. Prostate gene signatures. Table S2. Univariate analysis associating each signaling pathway with survival. Univariate cox proportional hazards model hazard ratios and unadjusted and adjusted P‐values for association of each signaling pathway with overall survival in the survival subset (n = 272). Table S3. Univariate analysis associating each genomic alteration with survival. Univariate cox proportional hazards model hazard ratios and unadjusted and adjusted P‐values for association of genomic alterations in AR, MYC, PTEN, RB1 and TP53 with overall survival in the survival subset (n = 272). [file MOL2-19-2348-s001.docx]

**Table S1. Prostate gene signatures.**

| Pathway | Reference | Category |
| --- | --- | --- |
| PTEN_loss_up_saal | (Saal et al., 2007) | AVPC |
| PTEN_loss_dn_saal | (Saal et al., 2007) | AVPC |
| RB1_loss_up_ertel | (Ertel et al., 2010) | AVPC |
| RB1_loss_dn_ertel | (Ertel et al., 2010) | AVPC |
| Neuro_1_FHCRC | (Labreque et al., 2019) | NEPC |
| Neuro_2_FHCRC | (Labreque et al., 2019) | NEPC |
| Neuro_All_FHCRC | (Labreque et al., 2019) | NEPC |
| AR_FHCRC | (Labreque et al., 2019) | CRPC subtype |
| SQUAM_FHCRC | (Labreque et al., 2019) | CRPC subtype |
| DNPC_FHCRC | (Labreque et al., 2019) | CRPC subtype |
| AR_L_FHCRC | (Labreque et al., 2019) | CRPC subtype |
| NE_FHCRC | (Labreque et al., 2019) | CRPC subtype |
| AMPH_FHCRC | (Labreque et al., 2019) | CRPC subtype |
| Lundberg_DNPC | (Lundberg et al., 2023) | CRPC subtype |
| Lundberg_AR_low | (Lundberg et al., 2023) | CRPC subtype |
| Lundberg_NE | (Lundberg et al., 2023) | NEPC |
| Lundberg_AR_NE | (Lundberg et al., 2023) | CRPC subtype |
| Beltran_NEPC_UP | (Beltran et al., 2016) | NEPC |
| Aggarwal_NEPC_UP | (Aggarwal et al, 2018) | NEPC |
| NELSON_RESPONSE_TO_ANDROGEN_UP | MDBSIG | AR signaling |
| WANG_RESPONSE_TO_ANDROGEN_UP | MDBSIG | AR signaling |
| Hieronymus_AR_signaling | (Hieronymus et al, 2006) | AR signaling |
| Zhang_basal | (Zhang et al, 2016) | Prostate subtype |
| Zhang_luminal | (Zhang et al, 2016) | Prostate subtype |
| Mu_Basal | (Mu et al, 2017) | Prostate subtype |
| Mu_luminal | (Mu et al, 2017) | Prostate subtype |
| RB1_loss_up_chen | (Chen et al, 2019) | AVPC |
| RB1_loss_dn_chen | (Chen et al, 2019) | AVPC |
| MetA | (Thysell et al, 2022) | CRPC subtype |
| MetB | (Thysell et al, 2022) | CRPC subtype |
| MetC | (Thysell et al, 2022) | CRPC subtype |

Abbreviations: Androgen Receptor (AR), Aggressive Variant Prostate Cancer (AVPC), Castrate Resistant Prostate Cancer (CRPC), Neuroendocrine Prostate Cancer (NEPC).

**Table S2. Univariate analysis associating each signaling pathway with survival.** Univariate cox proportional hazards model hazard ratios and unadjusted and adjusted p-values for association of each signaling pathway with overall survival in the survival subset (n=272).

| Pathway | HR | 95% CI | p-value | adjusted p-value and significance | |
| --- | --- | --- | --- | --- | --- |
| RB1_loss_up_ertel | 2.9 | [1.95-4.29] | 1.47e-07 | 3.19e-06 | **** |
| MetB | 2.4 | [1.75-3.42] | 1.43e-07 | 3.19e-06 | **** |
| HALLMARK_E2F_TARGETS | 3.2 | [2.09-4.97] | 1.07e-07 | 3.19e-06 | **** |
| PTEN_loss_up_saal | 3.9 | [2.3-6.53] | 3.39e-07 | 4.41e-06 | **** |
| HALLMARK_G2M_CHECKPOINT | 3.6 | [2.2-5.83] | 2.90e-07 | 4.41e-06 | **** |
| Aggarwal_NEPC_UP | 2.7 | [1.73-4.35] | 1.74e-05 | 1.88e-04 | *** |
| Mu_Luminal | 0.22 | [0.11-0.46] | 5.18e-05 | 4.81e-04 | *** |
| HALLMARK_DNA_REPAIR | 5.4 | [2.23-12.96] | 1.80e-04 | 1.30e-03 | ** |
| HALLMARK_MYC_TARGETS_V2 | 2.5 | [1.56-4.15] | 1.77e-04 | 1.30e-03 | ** |
| RB1_loss_up_chen | 3 | [1.66-5.35] | 2.67e-04 | 1.74e-03 | ** |
| HALLMARK_MYC_TARGETS_V1 | 2.4 | [1.45-3.92] | 6.06e-04 | 3.58e-03 | ** |
| HALLMARK_GLYCOLYSIS | 4.2 | [1.82-9.48] | 7.02e-04 | 3.80e-03 | ** |
| Zhang_luminal | 0.31 | [0.16-0.63] | 1.03e-03 | 5.15e-03 | ** |
| HALLMARK_MTORC1_SIGNALING | 2.8 | [1.44-5.54] | 2.56e-03 | 1.19e-02 | * |
| Hieronymus_AR_signaling | 0.41 | [0.22-0.75] | 4.08e-03 | 1.77e-02 | * |
| NELSON_RESPONSE_TO_ANDROGEN_UP | 0.46 | [0.25-0.86] | 1.51e-02 | 5.19e-02 | ns |
| MetA | 0.59 | [0.39-0.89] | 1.29e-02 | 5.19e-02 | ns |
| HALLMARK_ANDROGEN_RESPONSE | 0.46 | [0.25-0.86] | 1.50e-02 | 5.19e-02 | ns |
| HALLMARK_APICAL_SURFACE | 0.4 | [0.19-0.84] | 1.52e-02 | 5.19e-02 | ns |
| HALLMARK_MITOTIC_SPINDLE | 2.3 | [1.15-4.51] | 1.81e-02 | 5.88e-02 | ns |
| HALLMARK_OXIDATIVE_PHOSPHORYLATION | 1.9 | [1.1-3.14] | 2.02e-02 | 6.26e-02 | ns |
| HALLMARK_UNFOLDED_PROTEIN_RESPONSE | 2.5 | [1.15-5.49] | 2.12e-02 | 6.28e-02 | ns |
| PTEN_loss_dn_saal | 0.42 | [0.2-0.9] | 2.66e-02 | 7.53e-02 | ns |
| HALLMARK_CHOLESTEROL_HOMEOSTASIS | 2.1 | [1.08-4.22] | 2.82e-02 | 7.62e-02 | ns |
| Lundberg_AR_low | 0.45 | [0.22-0.92] | 2.95e-02 | 7.67e-02 | ns |
| Mu_Basal | 0.61 | [0.36-1.04] | 6.70e-02 | 1.67e-01 | ns |
| HALLMARK_HEDGEHOG_SIGNALING | 0.58 | [0.32-1.06] | 7.43e-02 | 1.79e-01 | ns |
| Neuro_2_FHCRC | 1.5 | [0.95-2.22] | 8.79e-02 | 1.97e-01 | ns |
| HALLMARK_IL6_JAK_STAT3_SIGNALING | 0.65 | [0.4-1.06] | 8.50e-02 | 1.97e-01 | ns |
| WANG_RESPONSE_TO_ANDROGEN_UP | 0.63 | [0.36-1.09] | 9.59e-02 | 2.02e-01 | ns |
| HALLMARK_INFLAMMATORY_RESPONSE | 0.62 | [0.35-1.09] | 9.63e-02 | 2.02e-01 | ns |
| HALLMARK_KRAS_SIGNALING_UP | 0.57 | [0.28-1.13] | 1.08e-01 | 2.19e-01 | ns |
| HALLMARK_ESTROGEN_RESPONSE_LATE | 2.2 | [0.81-5.88] | 1.21e-01 | 2.34e-01 | ns |
| HALLMARK_FATTY_ACID_METABOLISM | 1.8 | [0.85-3.98] | 1.22e-01 | 2.34e-01 | ns |
| RB1_loss_dn_chen | 0.57 | [0.28-1.19] | 1.34e-01 | 2.50e-01 | ns |
| MetC | 0.79 | [0.57-1.09] | 1.45e-01 | 2.61e-01 | ns |
| HALLMARK_IL2_STAT5_SIGNALING | 0.59 | [0.29-1.21] | 1.52e-01 | 2.67e-01 | ns |
| Beltran_NEPC_UP | 1.5 | [0.82-2.69] | 1.98e-01 | 3.38e-01 | ns |
| Neuro_All_FHCRC | 1.4 | [0.84-2.2] | 2.16e-01 | 3.42e-01 | ns |
| HALLMARK_INTERFERON_GAMMA_RESPONSE | 0.76 | [0.49-1.17] | 2.13e-01 | 3.42e-01 | ns |
| HALLMARK_KRAS_SIGNALING_DN | 0.42 | [0.11-1.65] | 2.13e-01 | 3.42e-01 | ns |
| SQUAM_FHCRC | 0.77 | [0.49-1.21] | 2.53e-01 | 3.92e-01 | ns |
| Zhang_basal | 0.73 | [0.43-1.27] | 2.66e-01 | 4.01e-01 | ns |
| Lundberg_AR_NE | 0.72 | [0.4-1.29] | 2.72e-01 | 4.01e-01 | ns |
| HALLMARK_WNT_BETA_CATENIN_SIGNALING | 0.73 | [0.38-1.4] | 3.39e-01 | 4.90e-01 | ns |
| AR_FHCRC | 0.81 | [0.5-1.31] | 3.82e-01 | 4.92e-01 | ns |
| HALLMARK_TNFA_SIGNALING_VIA_NFKB | 0.77 | [0.44-1.36] | 3.67e-01 | 4.92e-01 | ns |
| HALLMARK_NOTCH_SIGNALING | 0.75 | [0.39-1.42] | 3.72e-01 | 4.92e-01 | ns |
| HALLMARK_INTERFERON_ALPHA_RESPONSE | 0.84 | [0.56-1.25] | 3.86e-01 | 4.92e-01 | ns |
| HALLMARK_APICAL_JUNCTION | 0.74 | [0.39-1.43] | 3.74e-01 | 4.92e-01 | ns |
| HALLMARK_ANGIOGENESIS | 0.79 | [0.48-1.3] | 3.57e-01 | 4.92e-01 | ns |
| HALLMARK_PANCREAS_BETA_CELLS | 0.71 | [0.32-1.58] | 4.07e-01 | 5.09e-01 | ns |
| HALLMARK_REACTIVE_OXYGEN_SPECIES_PATHWAY | 1.3 | [0.66-2.59] | 4.38e-01 | 5.37e-01 | ns |
| Neuro_1_FHCRC | 1.1 | [0.76-1.68] | 5.54e-01 | 6.55e-01 | ns |
| Lundberg_NE | 0.8 | [0.38-1.66] | 5.45e-01 | 6.55e-01 | ns |
| HALLMARK_ESTROGEN_RESPONSE_EARLY | 0.8 | [0.3-2.14] | 6.57e-01 | 7.63e-01 | ns |
| HALLMARK_TGF_BETA_SIGNALING | 0.89 | [0.5-1.56] | 6.74e-01 | 7.69e-01 | ns |
| HALLMARK_HYPOXIA | 1.1 | [0.56-2.26] | 7.37e-01 | 7.85e-01 | ns |
| HALLMARK_APOPTOSIS | 0.89 | [0.46-1.72] | 7.31e-01 | 7.85e-01 | ns |
| HALLMARK_EPITHELIAL_MESENCHYMAL_TRANSITION | 0.93 | [0.6-1.42] | 7.27e-01 | 7.85e-01 | ns |
| HALLMARK_XENOBIOTIC_METABOLISM | 1.2 | [0.52-2.57] | 7.16e-01 | 7.85e-01 | ns |
| HALLMARK_PI3K_AKT_MTOR_SIGNALING | 0.86 | [0.31-2.34] | 7.67e-01 | 7.95e-01 | ns |
| RB1_loss_dn_ertel | 1.1 | [0.68-1.69] | 7.70e-01 | 7.95e-01 | ns |
| HALLMARK_P53_PATHWAY | 1.1 | [0.41-2.81] | 8.89e-01 | 9.03e-01 | ns |
| Lundberg_DNPC | 0.97 | [0.45-2.1] | 9.32e-01 | 9.32e-01 | ns |

**Table S3. Univariate analysis associating each genomic alteration with survival.** Univariate cox proportional hazards model hazard ratios and unadjusted and adjusted p-values for association of genomic alterations in *AR, MYC, PTEN, RB1* and *TP53* with overall survival in the survival subset (n=272).

| Alteration | HR | 95% CI | p-value | Adjusted p-value and significance | |
| --- | --- | --- | --- | --- | --- |
| RB1 2 copy loss | 2.6 | [1.59-4.32] | 1.63e-04 | 8.17e-04 | *** |
| MYC amplified | 1.6 | [1.07-2.32] | 2.05e-02 | 5.11e-02 | ns |
| TP53 2 copy loss | 1.5 | [1-2.33] | 5.16e-02 | 8.59e-02 | ns |
| PTEN 2 copy loss | 1.2 | [0.84-1.81] | 2.88e-01 | 3.60e-01 | ns |
| AR mutated or amplified | 1 | [0.68-1.54] | 9.16e-01 | 9.16e-01 | ns |
